# Bi-Material Negative Thermal Expansion Inverted Trapezoid Lattice based on A Composite Rod

**DOI:** 10.3390/ma12203379

**Published:** 2019-10-16

**Authors:** Weipeng Luo, Shuai Xue, Meng Zhang, Cun Zhao, Guoxi Li

**Affiliations:** College of Intelligent Science, National University of Defense Technology, Changsha 410073, Hunan, China; shuaixue1991@163.com (S.X.); z.mengdr@gmail.com (M.Z.);

**Keywords:** negative thermal expansion, bi-material, composite lattice, thermal, inverted trapezoid lattice

## Abstract

Negative thermal expansion (NTE) lattices are widely used in aerospace engineering where the structures experience large temperature variation. However, the available range of NTE of the current lattices is quite narrow, which severely limits their engineering application. In this paper, we report an inverted trapezoid lattice (ITL) with large NTE. The NTE of the ITL is 2.6 times that of a typical triangular lattice with the same height and hypotenuse angle. Theoretically, with a pin-jointed assumption, the ITL can improve the NTE by order of magnitude if the length ratio of the composite rod is changed. In the presented ITL, a composite rod is utilized as the base of the ITL. The composite rod has large inner NTE. The inverted trapezoid structure converts the inner NTE to the vertical direction contraction and obtains an extra NTE. Finite element simulations and experimental verification by interferometric measurement were conducted to verify the large thermal expansion of the ITL.

## 1. Introduction

Negative thermal expansion (NTE) structures have been successfully used in aerospace engineering and precision devices where the structures experience large temperature fluctuation [1,2]. For the new generation temperature-sensitive devices with lightweight, high-sensitivity and large measurement range, large NTE structures are especially required [3]. Bi-material triangular lattice is the most used NTE unit, due to its tailorable coefficient of thermal expansion (CTE) and lightweight. It requires extreme structural parameters to achieve large NTE. Its range of CTE is very narrow, considering the CTEs of the available materials and manufacturing capacity. This severely limits the development of the high-performance temperature-sensitive devices. Hence, proposing a larger NTE lattice is meaningful to improve the performance of temperature-sensitive devices.

Much work has focused on expending the magnitude of NTE [4,5,6]. Many solid materials with NTE have been fabricated, and the mechanisms of NTE have been researched, including tungstates [7,8,9], perovskites [10,11,12], magnetic nanocrystals [13], cyanide-bridged framework material [14] and samarium fulleride [15]. However, the densities of solid NTE materials are large. Another prevailing method is utilizing lightweight lattices to obtain large NTE, including topology optimization composites [2,16,17] and bi-material lattices [18,19,20,21,22,23,24]. These structures could achieve shrinkage in length or volume with temperature increase to obtain NTE. This NTE is just equivalent property, not a real material property. Among these structures, bi-material lattices are widely used in engineering, due to their excellent mechanical properties and a large range of CTE. These can be divided into two categories: Bending dominated lattice [25,26,27,28,29,30] and stretch-dominated lattice. The bending dominated lattice is based on the bi-material beam with initial curvature. The large stress between the joint interfaces and the complex manufacturing process limit the application of the lattices. The other category is stretch-dominated lattice, including planar lattices [19,20,21,22,31,32,33,34,35], cylindrical shell [36] and 3D bi-material lattices [37,38,39,40]. It uses a large thermal expansion of one material to generate an angle changes to generate NTE. Triangular lattice is the most used unit. Theoretically, it could obtain unbounded NTE. However, it requires extreme structural parameters (small height and large CTE ratio of the two constituents) to achieve large NTE. When the height of the triangular lattice becomes small, the number of lattices for the whole structure will increase. This makes the assembly difficult. On the other hand, the large CTE ratio of the two constituents will make large thermal stress in the joints. Thus, its range of CTE is very narrow, considering the CTEs of the available materials and assembly process. Fortunately, a promising approach employs the hierarchical structure to achieve large NTE [3,41,42]. The hierarchical structure uses small scale bi-material lattices to replace the elements in the origin lattice. However, the small scale bi-material lattice is hard to fabricate and assembly. Using the hierarchical structure alone can only enlarge the NTE for a small magnitude. In summary, design a novel lattice with larger NTE is challenging and meaningful.

In this paper, we report an ITL to overcome the limited NTE of conventional structures. The lattice has larger NTE compared with the bi-material triangular lattice with the same height and hypotenuse angle. In Section 2, we theoretically show that large inner NTE can be obtained by a composite rod. Based on the composite rod, we propose the ITL. In Section 3, finite element method analysis is conducted to reveal the influences of the structure parameters on the CTE and the maximum stress of the ITL. Moreover, comparisons of the ITL with the bi-material triangular lattice are conducted. In Section 4, CTE measurement by interferometry of the proposed lattice is performed to verify its excellent performance of large NTE. Discussions and conclusions are presented in Section 5 and Section 6, respectively.

## 2. The Composite Rod and the ITL

### 2.1. Composite Rod

#### 2.1.1. Structure of The Composite Rod

**Figure 1:**
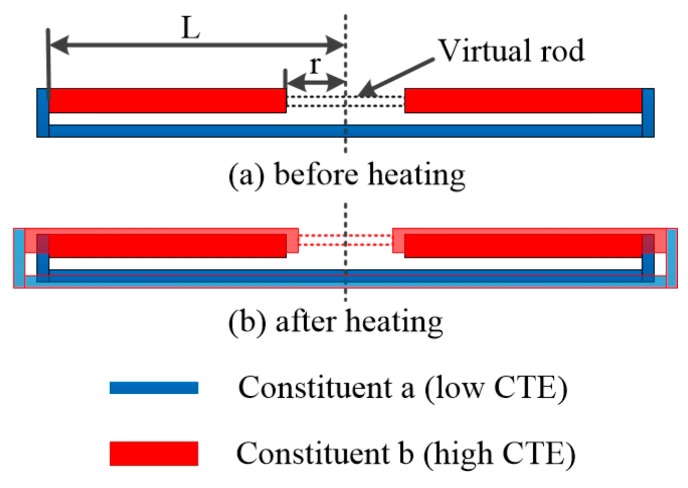
Schematic illustration of the composite rod. (**a**) Structure of the composite rod before heating. It consists of three rods, one long blue rod and two short red rods. The short rods have high coefficient of thermal expansion (CTE), while the long rod has low CTE. The ‘virtual rod’ is the part between the inner ends of the two short rods. ‘L’ is half the length of the long rod, and ‘r’ is half the length of the virtual rod. (**b**) Structure of the composite rod after heating. It shows the deformation mechanism of the composite rod after heating. The theoretical calculation shows that the ‘virtual rod’ could obtain a large magnitude of NTE.

The structure the composite rod is briefly presented here. It incorporates two constituents with different CTE. Constituent a with the lower CTE, α_a_, forms a long rod. Constituent b having the larger CTE, α_b_, is arranged as discontinuous short rod within the long rod, as shown in Figure 1. The part between the inner ends of the two short rods is the ‘virtual rod’. The ‘virtual rod’ could achieve large NTE if the length ratio L/r changes. It can be used as NTE rod for large NTE structure design.

#### 2.1.2. Thermal Expansion of the ‘Virtual Rod’

The thermal expansion of the virtual rod is determined by the origin length, the length variation and the temperature variation. The origin length of the structure is fixed. For a given temperature increment, the length change decides the equivalent thermal expansion of the virtual rod. In the composite rod, virtual rod’s CTE is decided by the thermal deformations of the long rod and the two short rods. For homogeneous temperature increment, the elongation of the long rod is smaller than that of the short rod. It generates a shrinkage of the inner ends of the two short rods. This will make the virtual rod with NTE. The ‘virtual rod’ has thermal expansion $\bar{\alpha }$ defined such that an increment in temperature ΔT causes a length change. With pin-jointed assumption, the detailed derivation of $\bar{\alpha }$ is given in Section S1 (Supplementary Materials),

(1) $$\bar{\alpha }=\frac{\alpha_{b}r-(\alpha_{b}-\alpha_{a})L}{r}.$$

**Figure 2:**
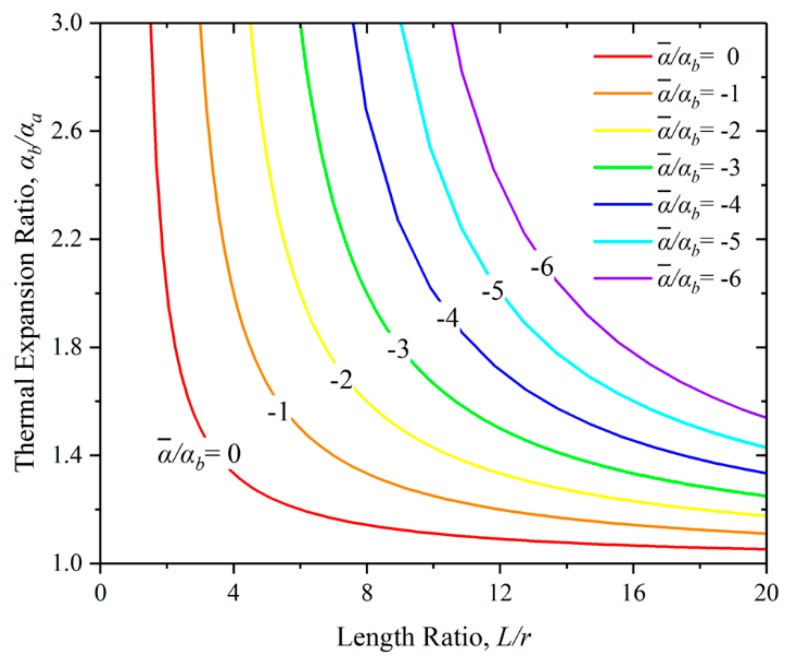
Contours of normalized net thermal expansion ratio $\bar{\alpha }$/α_b_ for a range of length ratio of the long rod and the virtual rod, L/r.

This relation between the thermal expansion ratio $\bar{\alpha }$/α_b_ and the length ratio L/r is plotted in Figure 2. For a given thermal expansion ratio, thermal expansion of the ‘virtual rod’ is increasing if the length ratio increases. It is apparent that the ‘virtual rod’ has negative net thermal expansion within a realizable window of length ratio and thermal expansion ratio. Specifically, when the parameters on the red line where α/α_b_ = 0, zero thermal expansion emerges.

There are two ways to achieve large inner NTE of the composite rod: Increasing the thermal expansion ratio and enlarging the length ratio. Large thermal expansion ratio will generate large thermal stress in the joints. On the contrary, it is easy to change the length ratio. The virtual rod’s NTE could be extremely large if the length ratio increases. The composite rod is like a negative multiplier. It converts the positive thermal expansion ratio, α_b_/α_a_, to negative thermal expansion ratio, α/α_b_. The large inner NTE composite rod lays the foundation for the ITL’s design.

### 2.2. ITL

The large NTE of the composite rod is only achieved at its inner ‘virtual rod’. The inner NTE is rarely used in engineering. Thus, we need to convert the inner NTE to the outer node. In this part, we design an ITL with large NTE in its vertical direction using the composite rod. It realizes this conversion.

#### 2.2.1. Structure of the ITL

**Figure 3:**
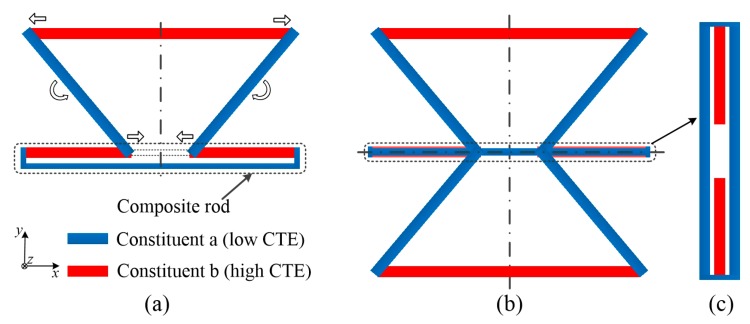
Schematic illustration of bi-material negative thermal expansion (NTE) inverted trapezoid lattices (ITLs). (**a**) Single ITL of anisotropic NTE. Constituent ‘a’ forms the hypotenuse, Constituent ‘b’ forms the upper base. The base is the composite rod. The two hypotenuses, upper base and the ‘virtual rod’ form an inverted isosceles trapezoid. The arrows show the deformation mechanism of the ITL after heating. (**b**) X-symmetric lattice of anisotropic NTE. The part in the dotted box is the composite rod. The composite rod is located in the xz plane. (**c**) Top view of the composite rod after 90° clockwise rotation.

The following features characterize the ITL. As shown in Figure 3a, the base is the composite rod. Constituent ‘a’ with the lower CTE, forms the hypotenuse. Constituent ‘b’ with the larger CTE, forms the upper base. The two hypotenuses, upper base and the ‘virtual rod’ form an inverted isosceles trapezoid. The topology could be connected by pin-jointed, welding or press-fit. It should enable the length changes to be accommodated by a rotation (angle change) at the nodes or deformation of the rods.

#### 2.2.2. NTE Mechanism of the ITL

For homogeneous temperature increment, thermal deformation of the lattice is determined by the thermal expansion of all the rods. As shown in Figure 3a, the arrows show the deformation mechanism of the ITL after heating. The base of the single ITL is the composite rod. It is negative deformation. The lower ends of the two hypotenuses move inward. While the upper base is positive thermal deformation. The upper ends of the two hypotenuses move outward. Then, the left hypotenuse rotates anticlockwise, and the right hypotenuse rotates clockwise. Thus, the inverted trapezoid structure converts the inner NTE of the composite rod to an extra vertical direction contraction through this rotation. It could enlarge the magnitude of NTE, due to the extra vertical contraction.

The assembly method must be decided ahead. The pin-jointed lattice allows free rotation at the nodes. It makes no stress in the lattice. However, the joint gaps at the nodes will generate remarkable CTE error. The rigid connection could overcome the former disadvantage. It could make the two constituents connect with no gap. This will decrease the CTE error of the ITL. Hence, the ITL lattices used in following are rigidly connected. With rigid connection assumption, the deformations of the rods have both thermal deformation and bending deformation. It is hard to theoretically figure out the thermal expansion of the ITL. Thus, the CTE and stress of the ITL are explored through finite element analysis.

## 3. Effects of The Basic Structure Parameters

In this section, finite element analysis was performed to explore the effects of the basic structural parameters of ITL by using COMSOL Multiphysics. Schematic illustration of the ITL is shown in Figure 3. With rigid connection assumption, the deformations of the rods are more complicated. The asymmetry of the structure will cause additional bending deformation. For a single ITL, as shown in Figure 3a, the upper base and the short rods of the base will have an extra bending deformation when the temperature rises. This is induced by the bending of the hypotenuse. The bending deformation will decrease the total NTE of the ITL. The X-symmetric ITL, as shown in Figure 3b, could overcome the extra bending deformation of the short rods of the composite rod. However, the two upper bases still have an extra bending deformation when the temperature rises. However, the ITL array could increase the structural symmetry. It could decrease the extra bending deformation. Thus, an ITL array model with three X-symmetric ITLs along the Y-direction was used in the following simulation.

**Figure 4:**
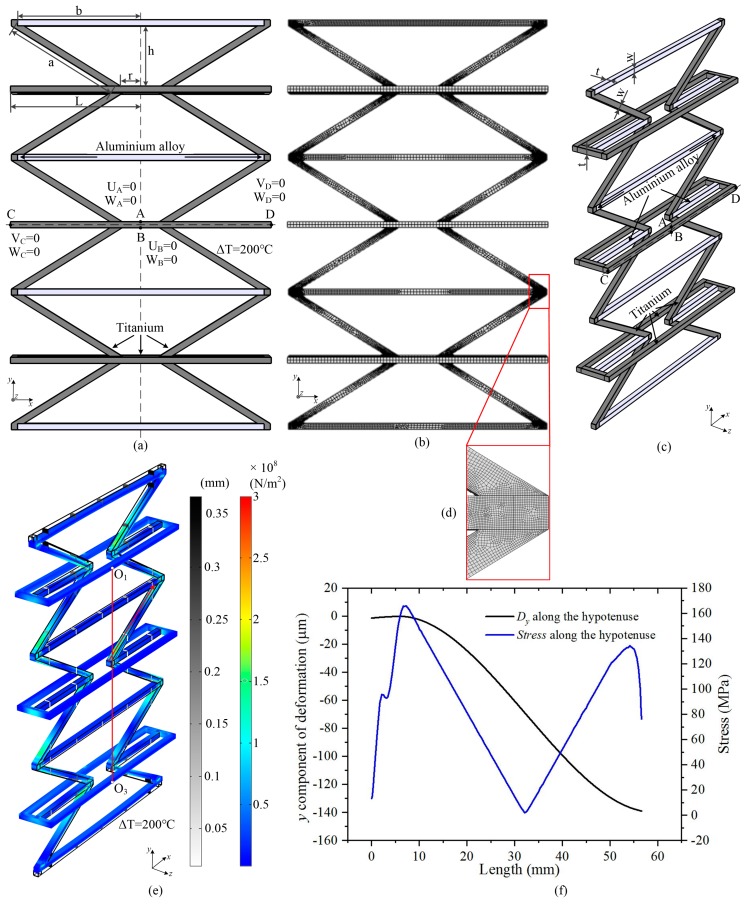
Geometrical model of the numerical simulation. (**a**) Front view of the geometrical model used in the numerical simulation and the boundary conditions in the model. (**b**) Finite element mesh of the ITL array. The members are meshed by hexahedral elements. (**c**) A 3D view of the geometrical model. (**d**) Finite element mesh of interface between the two constituents. (**e**) The deformation and stress of an ITL array (r = 24 mm, b = 60 mm, h = is 30 mm, L/r = 5, α_a_ = 8.6 × 10^−6^ K^−1^, α_a_ = 23 × 10^−6^ K^−1^, t = w = 5 mm) induced by a temperature increase of 200 ℃. The legend bars indicate overall deformation and stress distribution, respectively. The vertical red line is the center distance of the long rods on the first and third X-symmetric ITL. (**f**) The y component of deformation and stress along the hypotenuse. It is plotted according to the simulation results of the tilted red line in Figure 4 (**e**).

The geometrical model of an ITL array used in the numerical simulation is illustrated in Figure 4. The boundary conditions are illustrated in Figure 4a. It has three X-symmetric ITLs along the Y-direction. The two constituents are aluminium alloy and titanium, and the CTE ratio of the two constituents is ‘α_b_/α_a_’. Half the length of the long rod is ‘L’, half the length of the ‘virtual rod’ is ‘r’, the length of the hypotenuse of the isosceles trapezoid is ‘a’, half the length of the upper base is ‘b’, the height of the ITL is ‘h’. The 3D view of the geometrical model is show in Figure 4c. The width of all the rods are ‘w’; the thickness of all the rods are ‘t’. Due to the symmetry about y axis, the x-direction translation degree of freedom U for nodes A and B was restricted. Due to the symmetry about x axis, the y-direction translation degree of freedom V for nodes C and D was restricted. Besides, to avoid the z-direction translation, the z-direction translation degree of freedom W for nodes A, B, C and D was also constrained. With these constraints, the model was statically determined. In the simulation, the initial temperature was 20 ℃, and the final temperature was set as 220 ℃. As shown in Figure 4b, the members are meshed by free hexahedral elements. The maximum element size is 2.5 mm; the minimum element size is 0.25 mm. The number of the vertex elements is 262, the number of edge elements is 13988, the number of the boundary elements is 52620, the number of elements is 40584. In order to ensure a mesh independent result, the stress concentration areas are meshed by smaller element size to ensure quite dense mesh density, as shown in Figure 4d. Figure 4e shows the deformation and stress of an ITL array (r = 24 mm, b = 60 mm, h = is 30 mm, L/r = 5, α_a_ = 8.6 × 10^−6^ K^−1^, α_a_ = 23 × 10^−6^ K^−1^, t = w = 5 mm) induced by a temperature increase of 200 °C. The legend bars indicate overall deformation and stress distribution, respectively. The vertical red line is the center distance of the long rods on the first and third X-symmetric ITL. The equivalent NTE of the ITL was calculated according to the deformation of the center distance. Figure 4f provides the y component of deformation and stress along the hypotenuse. It was plotted according to the simulation results of the tilted red line in Figure 4e. The maximum stress of the bi-material ITL was calculated according to the curve of stress along the hypotenuse.

The following mainly studied the effects of the ITLs’ basic structural parameters, including the length ratio of the long rod and the ‘virtual rod’, ‘L/r’, the CTE ratio of the two constituents, ‘α_b_/α_a_’; and the height of the ITL, ‘h’. The two constituents used in the numerical simulation were aluminum alloy and titanium. Properties of the constituents are presented in Table 1. The properties of the materials did not change with the temperature during the simulation.

### 3.1. The Length Ratio of The Long Rod and The Virtual Rod, L/r

The length ratio of the long rod and the virtual rod determined the CTE of the composite rod. Therefore, the length ratio of the two rods could greatly affect the CTE of the bi-material ITL. In this part, the effects of the length ratio on the CTE and the maximum stress of the bi-material ITL were presented. An instructive comparison of the bi-material ITL and the traditional triangular lattice was made as well.

When the length ratio was changed from 2 to 10, CTE and stress of the ITLs were calculated. CTE was calculated by the Y-direction of the ITL, and the maximum stress was calculated without outer forces. The materials used in the numerical simulation were aluminum alloy and titanium. The other structural parameters except for the length of the long rod were fixed: The virtual rod length was 24 mm, the upper base length was 120 mm, the bi-material ITL height was 30 mm, the width of all the rods was 5 mm, the thickness of all the rods was 5 mm. The length ratio was changed by varying the length of the long rod. When the length ratio decreased to 1, the composite rod became only the long rod. Then, the two lower ends of the two hypotenuses will connect by the long rod. Thus, if L/r = 1, the Y-direction CTE the bi-material ITL would be equal to the Y-direction CTE of the traditional triangular lattice with the same height and hypotenuse angle.

**Figure 5:**
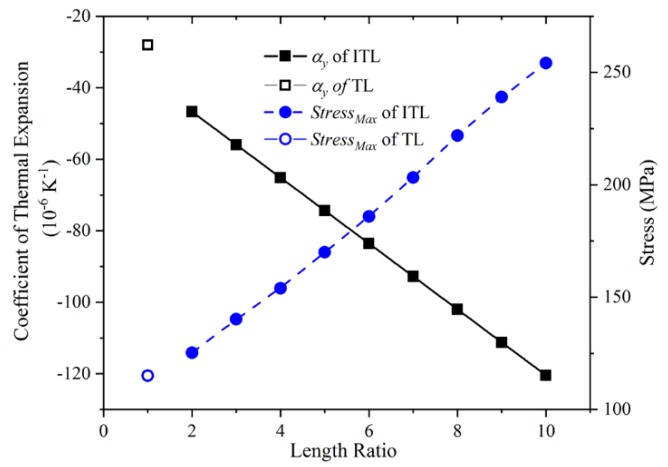
The Y-direction CTE and the maximum stress of the ITL with the varying length ratio L/r and these of the triangular lattice.

The Y-direction CTE and the maximum stress of the bi-material ITL are provided in Figure 5. The Y-direction CTE of the ITL linear decreased when the length ratio increases gradually. This is due to the CTE diminution of the composite rod. When the length ratio of the composite rod increases, the inner NTE of the composite rod will increase. The inner end of the short rod will push the hypotenuse to generate a larger angle of rotation. It generates a contraction along the vertical direction. Thus, the equivalent Y-direction CTE of the ITL linear decreases. In addition, the Y-direction CTE of the ITL is larger than that of the triangular lattice. This because the inner end of the short rod could push the hypotenuse to generate an extra rotation. It indicates the composite rod could enlarge the NTE of the ITL. On the other hand, the maximum stress of the ITL is linear increases when the length ratio increases. This is caused by the large deformation of the rod induced by the large NTE. It can be inferred that the ITL could achieve larger CTE compared with the triangular lattice when their height is equal.

### 3.2. The CTE Ratio of The Two Constituents

The CTE Ratio of the two constituents is the primary factors that must be decided at the first stage of the ITL designing. It is of great importance to analyze how the CTE ratio affects the Y-direction CTE and maximum stress of the ITL.

**Figure 6:**
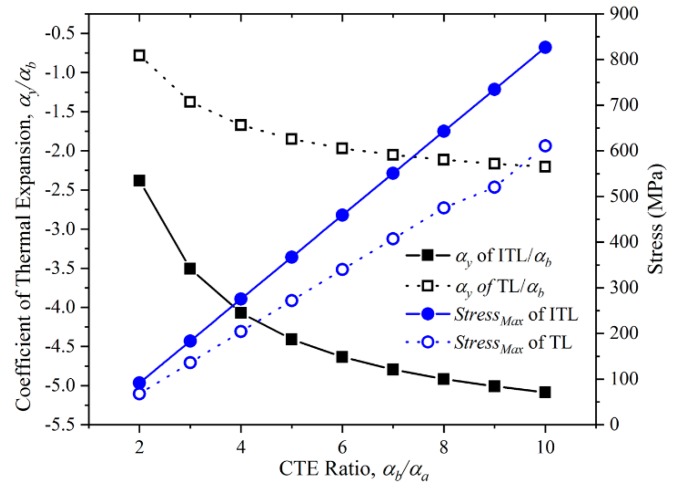
The Y-direction CTE and the maximum stress of the ITL with the varying CTE ratio of the two constituents and those of the triangular lattice.

CTE and maximum stress of the ITL were calculated when the CTE ratio of the two constituents ranges from 2 to 10. The CTE was calculated by the Y-direction of the ITL. In this case, we used the properties of aluminium alloy and titanium in the numerical simulation. The CTE of the titanium was fixed during the numerical simulation. The desired CTE ratio was achieved by varying the CTE setting of the aluminium alloy. The ratio of L/r was 5; the virtual rod length was 24 mm, the upper base length was 120mm, the bi-material ITL height was 30mm, the width of all the rods was 5 mm, the thickness of all the rods was 5 mm. The results are presented in Figure 6.

Figure 6 shows the Y-direction CTE and the maximum stress of the ITLs with different CTE ratio of the two constituents. The Y-direction CTE of the ITLs decreases as the CTE ratio of the two constituents increases. The gradient of the Y-direction CTE of the ITLs also decreases when the CTE ratio increases. The trend the Y-direction CTE of the triangular lattice is the same as that of the ITL. Large CTE ratio will generate large elongation of high CTE constituents. The large elongation will increase the rotation of the hypotenuse to enlarge the NTE. Moreover, the CTE of the ITL is more than two times compared with the CTE of the triangular lattice. This is not caused by the variation of the CTE ratio, but the composite rod. On the other hand, the maximum stress of the ITL increases when the CTE ratio increases. This is because the deformation of each rod increases as the CTE ratio increasing. Extremely large CTE ratio may lead to the maximum stress over the yield stress of the materials. Hence, it is advisable to choose a proper CTE ratio.

### 3.3. Height of The Lattice

**Figure 7:**
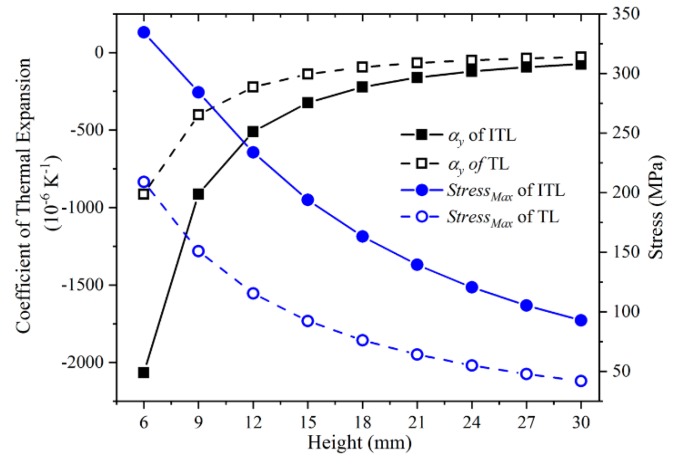
The Y-direction CTE and the maximum stress of the ITL with the varying height and those of the triangular lattice.

The height of the lattice was another important parameter. By changing the length of the hypotenuse and keeping the length of others rods constant, the height of the lattice changed. It had the same effect as changing the angle of the hypotenuse. It not only affected the CTE of the lattice, but also affected the stress and equivalent density. The Y-direction CTE and the maximum stress of the ITLs with the height varying from 6 to 30 mm were calculated. The properties of materials used in the simulation are provided in Table 1. Meanwhile, the ratio of L/r (5), the virtual rod length (24 mm), the upper base length (120 mm), the rod width (5 mm), the thickness of all the rods (5 mm) were fixed. The results are shown in Figure 7.

The Y-direction CTE and the maximum stress of the ITLs with different height are shown in Figure 7. The Y-direction CTE of the ITL and that of the triangular lattice both decrease when the height decreases. This is because the slant angle of the hypotenuse becomes larger if the height decreases. For the same displacement of the hypotenuse end, the larger slant angle, the larger vertical contraction. In addition, the Y-direction CTE of the ITL is over twice compared with that of the triangular lattice. This is induced by the composite rod. On the other hand, the maximum stress of the ITL and that of the triangular lattice both decrease when the height increases. This is because the Y-direction CTE become small. It makes the deformation of all the rods become small. While the maximum stress increases when the height decreases. This is due to the large rod deformation caused by the large CTE. 

It can be concluded that a reasonable height should be chosen. When the height decreases, the Y-direction CTE of the ITL decreases. Whereas, the equivalent density of the ITL increases. If the height of the lattice decreases to near zero, there will be not enough space for the negative thermal deformation. When the height is less than 6 mm, continuing to diminish the height would not be any good and the maximum stress increases quickly. It must ensure that the maximum stress less than the yield stress of the materials.

Through this simulation, the effects of the length ratio, CTE ratio and height are provided. These three parameters can change the NTE of the structure. As the height decreases, the NTE of the ITL increases sharply. However, the angle of the hypotenuse decreases with the height decreases. This will lead to a decrease in the bearing capacity of the ITL and large weight. The NTE increases faster when the CTE ratio range from 1–6, and the growth speed becomes slower and slower after it is greater than 6. The CTE ratio must be decided at the first stage of the ITL designing. It cannot be changed easily in following the process. These two parameters can be found in the traditional triangle lattice. The increase of length ratio makes the NTE almost linear increase. It makes the NTE of lattice larger and the design more flexible. The stress of structure is related to deformation. The greater the NTE, the greater the stress.

## 4. Experiment and Results 

**Figure 8:**
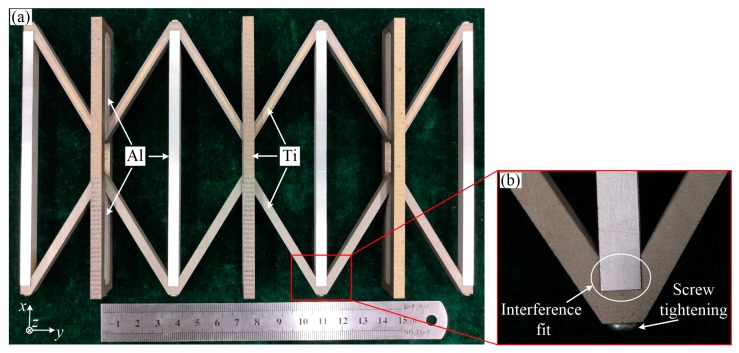
Images of the ITL sample. (**a**) Top view of the ITL lattice made of titanium (Ti TA2) and aluminium alloy (Al 7075-T6); (**b**) photograph of the joint. It uses an interference fit and fastens with screws.

In this section, large CTE of the ITL was verified through experiments. Two constituents were selected. They were the titanium (TA2) as constituent ‘a’ and the aluminium alloy (7075-T6) as constituent ‘b’. Over the range 20–200 °C, the average thermal expansions were 8.6 and 23.1 × 10^−6^ K^−1^ for the titanium and aluminium alloy. A combination of the two constituents fabricated the ITL array sample, as shown in Figure 8. The sample was the same as the model in the numerical simulation. It contained three X-symmetric ITL array along the Y-direction. The sample was manufactured by electric discharge machining from 5 mm thick plate of the constituents. The structure parameters of the sample are as follow. The ratio of L/r is 5; and the virtual rod length is 24 mm, the upper base length is 120 mm, the height is 30 mm, the rod width is 5 mm, the thickness of all the rods are 5 mm. As shown in Figure 8, it employs an interference fit with interference of 20 µm along X-direction. Figure 8b provides a photograph of the joint. Screw fastening was used to ensure the reliability of the connection.

**Figure 9:**
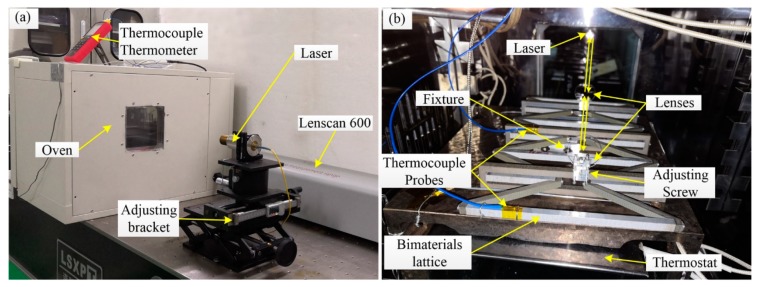
Experimental setup. (**a**) The overall diagram of the experimental apparatus, including Lenscan 600, laser, oven, adjusting bracket and thermocouple thermometer. (**b**) The experimental system inner the oven, including thermostat, thermocouple probes, bi-material lattice sample, two lenses, and adjusting the screw.

The displacement was measured between the long rods on the first and third X-symmetric ITLs, as shown in Figure 9b. It was measured by the Lenscan 600 (LS600, Nimes, France). It can measure the center thickness of optical elements and air gaps along the optical axis based on low coherence interferometry. Its measurement range was 600 mm, with an accuracy of ±1 µm. Two little lenses were assembled on the center of the long rods, the angle of the lenses could be adjusted by the adjusting screws, as shown in Figure 9b. Adjusted the height and position of the adjusting bracket of the laser to make the laser beam light on the lenses vertically. Then, adjusted the angle of the lenses to make the two reflected light spots coincide with the laser beam light. Thus, the light adjustment was completed.

**Figure 10:**
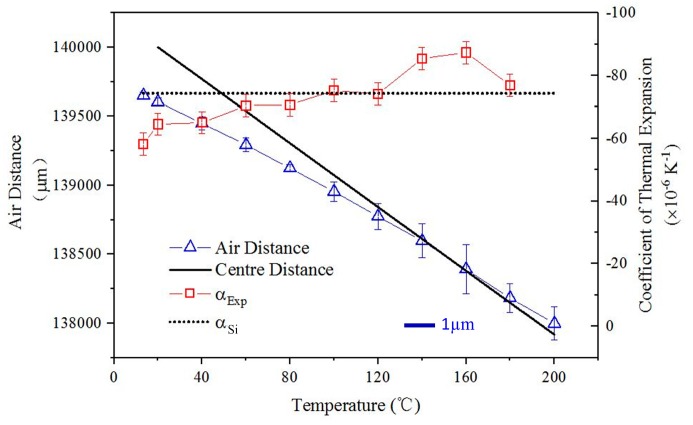
Thermal deformation measurement results of the X-symmetric ITL array. The air distance is the distance between the two lenses fixed on the sample. The center distance is the distance between the long rods on the first and third X-symmetric ITL in the simulation. The blue scale bar represents 1 µm in length of the measurement error of the air distance. The simulation CTE prediction is shown as a dotted line, while the experimental result is the solid line.

The specimen was heated slowly from 20 to 200 ℃ on a homemade hot plate. The specimen and the hot plate were put in an oven (101-0BS, Shanghai, China). The temperature of the hot plate was adjusted by the temperature control switch (HS-618F, Shanghai, China). The temperature of the specimen was measured using thermocouple probes located on the specimen in different positions, as shown in Figure 9b. The measurement value of the two thermocouple probes will indicate whether the specimen temperature is uniform or not. First, we set the oven and the hot plate to the target temperature. Second, when the temperature of the oven and the hot plate reach the target temperature, keep them at the target temperature for 30 min. Third, observe the measurement value of the two thermocouple probes. When the difference is less than 0.5 °C, measure the air distance between the two lenses. Then, increase the target temperature by 20 °C, repeat nine times. Finally, the Y-direction CTE of the array was calculated. The results are plotted in Figure 10, along with the numerical simulation predictions.

The air distance is the distance between the two lenses fixed on the center of the long rods. As shown in Figure 10, the air distance is decreasing when the temperature rises. It proves that the ITL array could achieve a Y-direction NTE. The simulation CTE prediction ‘α_Si_’ is shown as a dotted line, while the experimental CTE ‘α_Exp_’ is the solid line. The mean NTE of the sample is −74.4 × 10^−6^ K^−1^ from 20–200 °C. The experiment results indicate the ITL could achieve large value of NTE. Moreover, Figure 10 shows that the NTE of the sample increases gradually with the temperature rising. This because the Young’s modulus of the materials will decrease with the temperature rising. It will make the hypotenuse more easily to bending. Thus, the NTE of the sample increases gradually.

## 5. Discussion

According to the formula derived by Miller [21], the theoretical CTE of the triangular lattice was about −28 × 10^−6^ K^−1^ when the height and the angle of the hypotenuse were the same with the sample (see in Section S2 of Supplementary Materials). The experiment results showed the ITL based on the composite rod could achieve 2.6 times the value of NTE compared with the traditional triangular lattice. What is more, the factor will reach to 10 times when the length ratio of the L/r was 28 (see in section S2 of Supplementary Materials). The composite rod has great potential to be utilized to enlarge the magnitude of NTE of ITL. Moreover, the large NTE is independent of the size scale. By addressing the problem of isotropic NTE, connection and machining process, the composite rod and ITL can be used as elements for NTE structure design conveniently. 

The triangular lattice has been widely used to design various lattices. It could achieve a wide range of CTE and high stiffness. The prime advantage of our structure is utilizing a composite structure to enlarge the NTE when the constituents and height are restricted. Compared with the triangular lattice, the stiffness of the ITL is not enough. We will analyze and improve the stiffness of the ITL in future work.

The properties of the materials are not changed during the simulation. However, the Young modulus and the Poisson’s ratio could be temperature and stress-dependent. This will make the simulation results are inconsistent with the experimental results. The results in Figure 10 prove this different. Thus, the influences of temperature and stress on the properties of the materials should be considered in future work.

Following are considerations about future improving the measurement accuracy.

1. The residual stress of the sample has an important effect on the deformation measurement. During the machining process, we find that the remaining part of the plate after machining having a buckling deformation. The buckling deformation of the residual part of the plate indicates that there is residual stress on the sample. When the sample is heated, the residual stress on the specimen will redistribute. This will generate extra deformation and influence the measurement results. Heat treatment could obviously decrease the residual stress. Hence, we decrease the measurement error thought heat treatment.
2. The temperature homogeneity is another factor which influent the thermal deformation of the sample. When the sample is heated, the temperature of the part, which is close to the heat source, is higher compared with the other part. In the experiment, to obtain an approximate homogeneous temperature, we used the thermocouple probes to monitor the temperature of the sample in different positions. It can only provide the temperature of the measurement point. This is not consistent with the homogeneous temperature hypothesis. It will generate a large measurement error. In our future work, we will use the thermal imager to monitor the temperature of the sample. It will provide a global temperature distribution of the sample. This will decrease the temperature differences of the different part of the sample, and improve the accuracy of the measuring results.
3. The air refractive index also affects the measurement results. It needs the air refractive index to calculate the optical distance of the two parallel lenses. During the measurement, the air refractive index is set as a constant, but it changes when the temperature increases actually. It should be considered in order to obtain a more accurate results in our future work.

## 6. Conclusions

In this paper, we reported an ITL based on the composite rod. The inverted trapezoid structure realized that the inner NTE of the composite rod converted to an extra vertical NTE of the ITL. The magnitude of NTE of the proposed lattice was analyzed numerically and experimentally. The finite element simulations showed that larger NTE could be achieved by increasing the length ratio, increasing the CTE ratio of the two constituents, or decreasing the height of the ITL. The NTE of ITL was better compared with the traditional triangular lattice. It provided an explicit guideline for designing an ITL with large NTE. The ITL specimen was fabricated by the electric discharge machining. The CTE measurement results by the laser interferometry successfully demonstrated its large NTE. NTE of the ITL is 2.6 times, compared with the traditional triangular lattice. Theoretically, the ITL could improve the NTE by order of magnitude if the length ratio of the composite rod was large enough. This structure is especially useful for temperature-sensitive sensors with high-sensitivity and thermal actuator with a large stroke.

## Figures and Tables

**Table 1:** Material properties for the aluminum alloy [43], and titanium [44].

Material	Young’s Modulus E (GPa)	Coefficient of Thermal Expansion α (×10^−6^ K^−1^)
Al alloy 7075-T6	72	23.1
Ti TA2	107.9	8.6

## Supplementary Materials

The following are available online at https://www.mdpi.com/1996-1944/12/20/3379/s1, Section S1: Thermal expansion of the composite rod, Section S2: Thermal expansion calculation.
