# Supplementary material for: Bi-Material Negative Thermal Expansion Inverted Trapezoid Lattice based on A Composite Rod
Source: Materials (Basel). 2019 Oct 16;12(20):3379. doi: 10.3390/ma12203379 (PMC6829452; doi:10.3390/ma12203379)
Supplement: Supplementary file 1 [file materials-12-03379-s001.pdf]

materials

Supporting information

Bi-Material Negative Thermal Expansion Inverted Trapezoid Lattice based on A Composite Rod

Weipeng Luo *, Shuai Xue, Meng Zhang, Cun Zhao, Guoxi Li *

College of Intelligent Science, National University of Defense Technology, Changsha, Hunan, China; shuaixue1991@163.com (S.X.); z.mengdr@gmail.com (M.Z.); zhaocun18@163.com (C.Z.)
* Correspondence: luoweipeng09@nudt.edu.cn (W.L.); lgx2020@sina.com (G.L.)

S1. Thermal Expansion of The Composite Rod

The virtual rod's coefficient of thermal expansion (CTE) is decided by the thermal deformation of the long rod and the two short rods. For homogeneous temperature increment, the elongation of the long rod is smaller than that of the short rod, which generates shrinkage of the inner ends of the two short rods. This make the virtual rod with negative thermal expansion (NTE). The virtual rod has thermal expansion $\overline{\alpha}$ defined such that an increment in temperature ΔT causes a length change. The formula for calculating the equivalent CTE of the virtual rod is given as follows.

For an increment in temperature ΔT, the elongation of the long rod is D_L, the elongation of the short rod is D_S, and the elongation of the virtual rod is D_V, thus

$$D_L = 2\alpha_a \cdot L \cdot \Delta T \,, \tag{1}$$

$$D_S = \alpha_b \cdot (L\text{-}r) \cdot \Delta T \,, \tag{2}$$

$$D_V = D_L - 2D_S \,, \tag{3}$$

and in general:

$$\overline{\alpha} = \frac{D_V}{2r\Delta T} \,. \tag{4}$$

Therefore, combining the equations:

$$\overline{\alpha} = \frac{\alpha_b r - (\alpha_b - \alpha_a)\ L}{r} \,. \tag{5}$$

S2. Thermal Expansion Calculation

2.1. Thermal Expansion of The Bi-Materials Triangular Lattice

The parameters of the inverted trapezoid lattice (ITL) are as follows. The ratio of *L*/*r* is 5, the virtual rod length is 24 mm, the upper base length is 120 mm, the height is 30 mm, the rod width is 5 mm, and all rods have a thickness of 5 mm. The parameters of the triangular lattice are the same as with the ITL, as shown in Figure S1. Thus, the angle of the hypotenuse and the height of the lattice are the same. The two constituents are aluminum alloy and titanium.

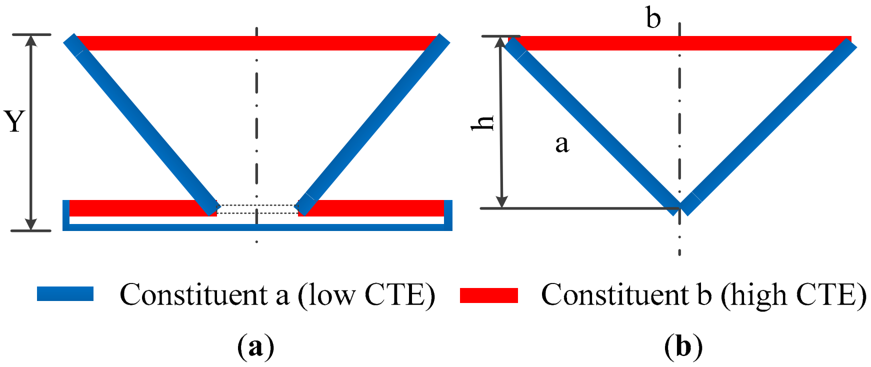

Figure S1. Geometrical structure illustrations of the two lattices. (**a**) Schematic illustration of the ITL. (**b**) Schematic illustration of the bi-material triangular lattices. The height and the base angle are the same. CTE: coefficient of thermal expansion.

According to the CTE equation of a triangular unit, which was derived by Miller [1], the thermal expansion of the *y*-direction, α_y, is

$$\alpha_y = \frac{a^2\alpha_a - \frac{b^2}{4}\alpha_b}{h^2}. \quad (6)$$

When α_a = 8.6 × 10⁻⁶ K⁻¹, α_b = 23.1 × 10⁻⁶ K⁻¹, $a = \sqrt{b^2/4 + h^2}$ = 56.6 mm, according to Equation (6), the thermal expansion of the triangular lattice is $\alpha_y = (a^2\alpha_a - b^2/4 * \alpha_b)/h^2 = -28.5 \times 10^{-6}$ K⁻¹.

2.2. *Thermal Expansion of The Bi-Materials Inverted Trapezoid Lattice with The Varying Length Ratio*

It is difficult to theoretically figure out the CTE of the ITL with a rigid connection assumption. However, it can be approximated with a pin-jointed assumption. According to the CTE calculation method of Miller [1], considering that the rods of the ITL can freely rotate at the nodes, the thermal expansivities along the *y*-axis are a function of the geometry of ITL. The thermal expansivity of the ITL can be calculated by considering half of the isosceles trapezoid to form a Pythagorean triangle, thus:

$$y^2 = a^2 - (b-r)^2, \quad (7)$$

$$(y+\delta y)^2 = (a+\delta a)^2 - [(b+\delta b)-(r+\delta r)]^2, \quad (8)$$

and in general:

$$\alpha_y = \frac{\delta y}{y\delta T}. \quad (9)$$

Therefore, combining Equations (7)–(9), the thermal expansion of the bi-material inverted trapezoid lattices along the *y*-axis direction is given as:

$$\alpha_y = \frac{a^2\alpha_a - (b-r)^2\alpha_b - (b-r)L(\alpha_b - \alpha_a)}{a^2 - (b-r)^2}. \quad (10)$$

The following calculation provides the results of CTE with the length ratio changes. The CTEs of ITL are calculated according to Equation (10). The CTEs used in the calculation are the property of the two constituents: aluminum alloy and titanium. The other structural parameters except the length of long rod are fixed: the virtual rod length is 24 mm, the upper base length is 120 mm, the bi-material ITL height is 30 mm, and all rods are 5 mm wide and 5 mm thick. The length ratio is changed by varying the length of the long rod. The results are presented in Table S1.

Table S1. Theoretical *y*-direction CTE of the ITL with the varying length ratio *L*/*r*.

Length Ratio	CTE (10^{-6} K^{-1})
2	−46.7
3	−55.9
4	−65.1
5	−74.3
6	−83.6
7	−92.8
8	−102.0
9	−111.2
10	−120.4
11	−129.6
12	−138.9
13	−148.1
14	−157.3
15	−166.5
16	−175.7
17	−184.9
18	−194.2
19	−203.4
20	−212.6
21	−221.8
22	−231.0
23	−240.2
24	−249.4
25	−258.7
26	−267.9
27	−277.1
28	**−286.3**
29	−295.5
30	−304.7

With length ratio increasing, the NTE of the bi-material inverted trapezoid lattices increases continuously. Considering that the CTE of the triangular lattice is -28.5×10^{-6} K^{-1} (Section S2.1), when the length ratio is 28, the NTE of the bi-material inverted trapezoid lattices is -286.3×10^{-6} K^{-1}. Thus, the NTE of the bi-material inverted trapezoid lattices will reach to 10 that of the traditional triangular lattice.

It is easy to achieve a large length ratio by changing the length of the long rod. However, there are still some restricting factors in practical applications.

1. Buckling.

With a large length ratio, the length of the short rod will become long. Then, the short rod becomes slender and unstable under compressive stress. This will make the short rod buckle and lead to structural failure. The pin-jointed lattice allows free rotation at the nodes. It makes no stress in the lattice. This connection is a possible solution to the buckling. However, the joint gaps at the nodes will generate remarkable CTE error.

2. Geometrical limits.

First, when the length ratio is large enough, the inner end of the short rod will meet together as temperature increases. This will restrict the negative thermal expansion of the virtual rod and generate large stress.

Second, large length ratio makes the structure’s ratio of length to width seriously out of balance.

Reference

1. Miller, W.; Mackenzie, D.S.; Smith, C.W.; Evans, K.E. A generalised scale-independent mechanism for tailoring of thermal expansivity: Positive and negative. *Mech. Mater.* **2008**, *40*, 351–361.

© 2019 by the authors. Submitted for possible open access publication under the terms and conditions of the Creative Commons Attribution (CC BY) license (http://creativecommons.org/licenses/by/4.0/).
